# Rotator cuff tear with concomitant long head of biceps tendon (LHBT) degeneration: what is the preferred choice? Open subpectoral versus arthroscopic intraarticular tenodesis

**DOI:** 10.1186/s10195-019-0531-5

**Published:** 2019-07-05

**Authors:** Mohsen Mardani-Kivi, Sohrab Keyhani, Mohammad-Hossein Ebrahim-Zadeh, Keyvan Hashemi-Motlagh, Khashayar Saheb-Ekhtiari

**Affiliations:** 1 Orthopedic Department, Guilan University of Medical Sciences, Parastar Ave, Poursina Hospital, P.O. Box: 4193713191, Rasht, Iran; 2 Orthopedic Department, Shahid Beheshti University of Medical Sciences, Tehran, Iran; 3 Orthopedic Department, Mashhad University of Medical Sciences, Mashhad, Iran

**Keywords:** Rotator cuff tear, Open subpectoral tenodesis, Arthroscopic intraarticular tenodesis, Long head of biceps tendon, Shoulder function, Pain intensity

## Abstract

**Background:**

Lesions associated with the biceps tendon are commonly detected during arthroscopic repair of rotator cuff tears. Acquiring a preferable technique to repair both cuff and long head of biceps tendon (LHBT) lesions was the aim of several recent studies. This study aimed to compare clinical and functional outcomes of open subpectoral versus arthroscopic intraarticular tenodesis in patients with repairable rotator cuff tear associated with LHBT degeneration.

**Patients and methods:**

In this randomized clinical trial, 60 eligible candidates for arthroscopic rotator cuff repair (mean age 55.7 ± 6.9 years) were allocated to a control group (open subpectoral, SP) or intervention group (intraarticular, IA). In the IA group, an anchor suture was used for both rotator cuff repair and LHBT tenodesis. In the SP group, after arthroscopic repair of the rotator cuff, subpectoral tenodesis of LHBT was performed using an interference screw. Patients were evaluated for 2 years follow-up regarding pain intensity using the visual analogue scale (VAS) and shoulder function using the Constant Score and Simple Shoulder Test.

**Results:**

The two groups were similar with regard to demographic characteristics and preoperative evaluations (all *P* > 0.05). The functional status of both groups was improved, but not significantly differently so between the two groups (*P* = 0.1 and *P* = 0.4, respectively). Pain intensity decreased during the 2-year follow-up period, similarly so in the two groups. Patient satisfaction was also similar in the two groups.

**Conclusion:**

Large and massive rotator cuff tears (tears > 3 cm) associated with LHBT pathologies benefited from intraarticular or subpectoral tenodesis similarly, with no differences in short- or mid-term results between these two techniques.

**Level of evidence:**

II.

## Introduction

Two-thirds of patients with long head of biceps tendon (LHBT) pathology have simultaneous rotator cuff tear, which may lead to anterior shoulder pain and forward flexion dysfunction [1–3]. Rotator cuff tear is a debilitating condition and plays a crucial role in determining health status according to the 36-Item Short Form (SF-36) questionnaire [4–6]. Streit et al. [7] showed that biceps involvement was not a chronic or acute inflammatory process from a pathological view, but exhibited myxoid and degenerative changes similar to the pathological findings of De Qeurvain syndrome. Therefore, the term “tendinosis” is a clearer and improved description rather than “tendinitis.” There are several therapeutic approaches ranging from conservative methods to open surgical or arthroscopic approaches [6–11] based on the type of lesion [5]. After demonstrating that releasing the LHBT may alleviate pain even in an irreparable rotator cuff tear, a wide range of surgical methods were proposed. The two forerunner surgical methods are tenotomy and tenodesis; the effectiveness of these was evaluated in previous reports [3, 12–18]. Tenotomy is relatively simple and reproducible with reduced healing time after surgery [7, 12, 16]. Tenodesis is a more demanding procedure with longer operative and recovery times, but offers some theoretical advantages over tenotomy, including lack of cosmetic problems such as Popeye sign, maintaining supination and elbow flexion power, avoiding cramping pain, and preventing muscle atrophy [4, 7]. However, recent studies [3, 6, 7, 15, 16] showed similar therapeutic results for the two techniques.

A majority of surgeons prefer tenotomy for elderly patients (> 65 years of age) but tenodesis for younger patients with a higher level of activity, workers, and athletes [11, 19, 20]. In general, tenodesis techniques can be categorized based on the type of surgery (open, mini-open, and arthroscopic), fixation site (proximal or distal to the bicipital groove), and fixation type (interference screw, bone tunnel, suture anchors, and keyholes) [4, 7]. Open subpectoral tenodesis (SP) [21, 22] and arthroscopic intraarticular tenodesis (IA) [23] are the most popular techniques.

Interestingly, the number of studies with random assignment evaluating tenodesis with rotator cuff tear repair is extremely limited [4, 7, 24]. The aim of the present study is to evaluate and compare functional and clinical results of open SP and arthroscopic IA in repairable rotator cuff tear with concomitant LHBT degeneration. The null hypothesis is that the therapeutic and clinical outcomes of these two methods are similar.

## Materials and methods

### Sample size calculation

The sample size required for comparison of the first outcome between the two groups was determined based on De Carli [12] with 95% confidence and 95% power in two-way analysis of statistical difference. Considering 10% sample loss, a sample size of 30 persons in each group was determined. Intent-to-treat analysis was applied to compare the primary and secondary outcomes between the IA (30 cases) and SP (30 cases) groups.

### Patients

The study was in accordance with the Consolidated Standards of Reporting Trials (CONSORT) statement. In this randomized clinical trial study, all patients who were candidates for arthroscopic repair of rotator cuff tear (large or massive, tear > 3 cm) with anterior shoulder pain, at least one positive biceps test (speed test, Yergason’s test, and active compression or biceps instability test), and who also had subluxation, dislocation, partial tear, or superior labral tear from anterior to posterior (SLAP) lesion on arthroscopic evaluations were enrolled. The inclusion criteria were age 18 to 65 years and finding no evidence of extensive fatty infiltration in ruptured rotator cuff muscles on magnetic resonance imaging (MRI). Patients with history of shoulder surgery, tumors or cysts in the area of the bicipital groove and the proximal humeral shaft, pain in both shoulders, and impossibility of arthroscopic rotator cuff tear repair during surgery and conversion to open surgery were excluded from the study.

### Surgical techniques

SP [21, 22] is considered to be the more common technique, and was therefore considered to be the control technique. IA [23] was considered to be the intervention group. Eligible patients were randomly allocated to one of the two groups with the help of the random block method with four patients in each block. General anesthesia was performed in all patients while in the beach chair position. Conditions of the shoulder joint, level of rotator cuff, and biceps tendon lesions were visualized via a lateral port. The rotator cuff was repaired with a suture anchor number 5 mm (Arthrex Inc., Naples, FL, USA). The same suture was also crossed along the damaged biceps tendon. LHBT was tenodesed to greater tuberosity, and it was detached from the glenoid. In the SP group, a 2-cm incision was created distal to the pectoral major muscle and, after crossing the guidewire, the biceps tendon was tenodesed to the bicipital groove using an interference screw of appropriate size (Arthrex Inc., Naples, FL, USA).

### Rehabilitation protocol

Rehabilitation after surgery was similar in both groups. In the first 6 weeks after surgery, a sling with an abduction pad was used. Active flexion and extension of the elbow were allowed, but terminal extension was forbidden. After 6 weeks, the sling was removed. Isotonic strengthening of the fixator muscles of the rotator cuff, deltoid muscle, and scapula was started 10–12 weeks postoperatively. This rehabilitation protocol was continued for 6 months in both groups. Heavy manual work and overhead activities were allowed only after sufficient muscle strengthening at approximately 6–10 months after surgery.

### Measurements

The primary outcome was shoulder function based on Constant Score and Simple Shoulder Test. Secondary outcomes were pain intensity based on the visual analogue scale (VAS) score before and at 6 and 24 months after surgery. In the last follow-up visit, patients were able to express their satisfaction with the results of the treatment based on a VAS score from 0 to 10.

### Statistical analysis

Collected data were statistically analyzed using SPSS version 19.0 for Windows (SPSS Inc., Chicago, IL, USA). Qualitative and quantitative data were analyzed by chi-square and independent *t* test, respectively. Analysis of variance (ANOVA) tests were used for analysis of Constant Score, SST, and VAS scores based on normality of data. For comparison of disease trend after treatment, repeated-measures analysis was used. *P* < 0.05 was considered to be significant.

## Results

**Fig. 1:**
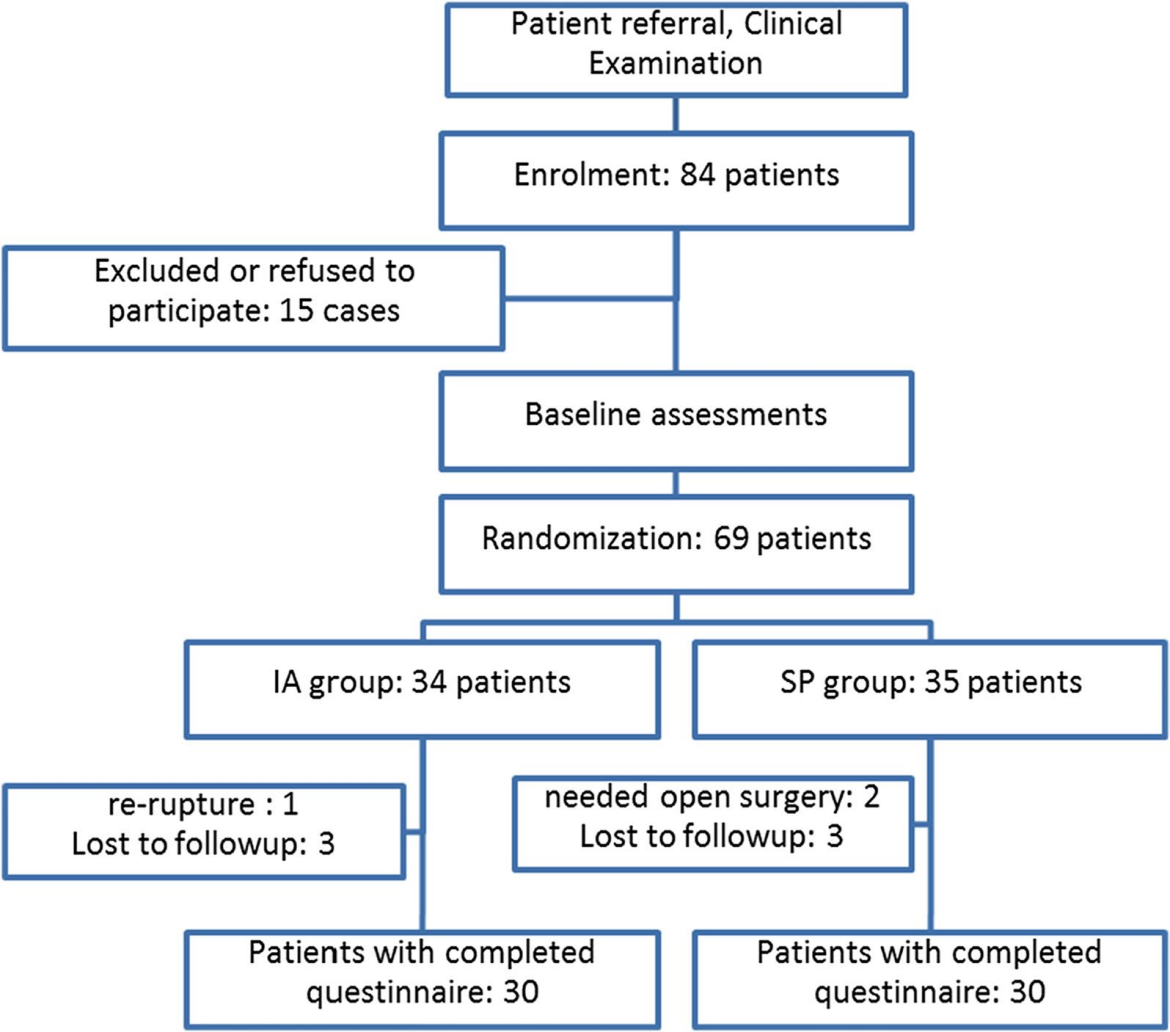
Study flowchart

Initially, 84 patients were eligible for the study, but 15 patients did not meet the inclusion criteria and were eliminated from participation. Of the 69 remained patients, 34 patients were in the IA group, and 35 patients were in the SP group. Two patients (both in the SP group) also needed open surgery during arthroscopic surgery for repair of the rotator cuff, and were therefore excluded from the study. One patient in the IA group had rerupture of the rotator cuff 1.5 years after acute trauma, and in this procedure, rotator cuff repair and subpectoral tenodesis were applied, excluding the patient from the study. Six patients (three in each group) were lost to follow-up and, therefore, were excluded (Fig. 1).

The two groups showed similar demographic characteristics (age and gender) and acromion type frequency (*P* > 0.05) (Table 1). Patients were similar regarding preoperative scores (Constant, SST, and VAS scores) (*P* > 0.05). Repeated-measures analysis (Greenhouse–Geisser) showed that significant change occurred over time (*P* < 0.001). As shown in Table 2, Constant Score and SST increased in both groups. This means that patients in both groups had improvement, but the improvement in the IA group was not better than that in the SP group (*P* = 0.1 and *P* = 0.4, respectively). Pain intensity based on VAS score also decreased significantly during follow-up in both groups (*P* < 0.001). However, both techniques decreased pain intensity at a similar level (*P* = 0.3). At final follow-up visit, the patient’s satisfaction showed no significant difference between the IA and SP groups (9.7 ± 0.5 and 9.5 ± 0.7 respectively, *P* = 0.47).

**Table 1:** Demographic characteristics and baseline assessments

	IA	SP	Both	Statistics
No.	30	30	60	–
Age (mean ± SD, years)	56.1 ± 6.2	55.2 ± 7.7	55.7 ± 6.9	N.S^a^
Sex (male/female)	12/18	14/16	26/34	N.S^b^
Acromion type (III/II/I)	(20/7/3)	(18/9/3)	(38/16/6)	N.S.^b^
Dominant hand (right/left)	23/7	26/4	49/11	N.S^b^
Affected hand (right/left)	20/10	21/9	41/19	N.S^b^

*SD* standard deviation, *IA* intraarticular, *SP* subpectoral, *N.S.* nonsignificant

^a^Independent-sample *t* test

^b^Chi-square test

**Table 2:** Primary (Constant and SST scores) and secondary (VAS score) outcomes in both groups

	Preoperative		At 6-month follow-up		At 2-year follow-up	
	IA	SP	IA	SP	IA	SP
Constant (mean ± SD)	54.2 ± 6.1	52.1 ± 7.2	82.1 ± 5.6	81.2 ± 6.9	93.1 ± 3.9	92.7 ± 5.2
SST (mean ± SD)	3.9 ± 0.8	3.4 ± 0.8	10.2 ± 0.7	10.2 ± 0.8	11.5 ± 0.7	11.3 ± 0.8
VAS (mean ± SD)	8.2 ± 1	8.4 ± 1.1	2 ± 0.8	2.2 ± 0.9	0.4 ± 0.6	0.4 ± 0.5

*SST* Simple Shoulder Test, *VAS* visual analogue scale, *SD* standard deviation, *IA* intraarticular, *SP* subpectoral

## Discussion

The present study confirmed that the type of surgery did not result in significant differences in the outcomes of LHBT lesions with rotator cuff tear treatment. We found that the IA method is as effective as the SP method in terms of both therapeutic outcomes and patient satisfaction.

In the Mazoka study, open subpectoral with bone tunnel, open subpectoral with screw, arthroscopic with screw, and arthroscopic with suture anchor techniques were compared with regard to biomechanical properties [11]. They reported that these four techniques showed no significant differences. In another study, Abraham and colleagues evaluated the open or arthroscopic surgical technique in a systematic review [8]. After evaluation, they found that 98% of patients had good to excellent final results and that the type of surgery, open or arthroscopic, had no significant effect on the treatment response. Gombera et al. and Werner et al. [23, 25] evaluated the therapeutic results of treatment of isolated lesions of LHBT by open subpectoral versus arthroscopic methods, finding no significant differences between these two methods in isolated biceps tendon lesions. Based on these studies, it can be suggested that both methods provide promising results in the treatment of LHBT lesions, either with or without rotator cuff tear.

One may say that, with similar results, arthroscopic techniques may be slightly, but not significantly, favorable over other methods. Using arthroscopic methods, there is less damage of soft tissue and concurrent lesions can be treated using one approach. Meanwhile, in the open subpectoral method, two approaches and one additional incision are needed, resulting in more damage to soft tissue and longer recovery. Furthermore, the subpectoral method requires an interference screw for tenodesis, thus an additional financial burden may be imposed on patients.

However, there are several reported advantages of the subpectoral technique, including more effective visualization during surgery. It has been shown that mean tendon visualization by the arthroscopic method is just 32%, and 56% of patients with LHBT pathologies may be underestimated accordingly [17]. The subpectoral method is also able to reliably maintain the anatomic length–tension relationship of the biceps muscles by placing the musculotendinous junction of the biceps at the inferior border of pectoralis major tendon [23]. Heckman et al. [19] believe that the subpectoral approach is the most reasonable technique for revision tenodesis when pain persists due to mechanical failure or mistreatment of other shoulder pathologies in previous surgery. We also used this technique for one patient who had rerupture due to another acute trauma.

Another remaining concern with arthroscopic methods relates to the fixation site of LHBT. Some believe that, if the tendon is fixed too proximally in the bicipital groove, it may produce consistent pain and chronic tendinopathies. This issue was evaluated in a recent cadaveric study by Johannsen et al. [20], which demonstrated that both methods could fix the tendon at the proper distal site of the groove. However, the mean of the tendon fixation site in the subpectoral method was 2.2 cm more distal than in the arthroscopic approach [20]. It has been shown that there is a vast sensory neural plexus in the biceps tendon and that their innervations come from the proximal site of tendon; for this reason, subpectoral tenodesis may also decrease the pain stimuli along with tendon fixation and prevention of impingement related to tendon attachment to the proximal bicipital groove [11]. The clinical outcomes of surgeries for these pathologies (cuff tear plus biceps tendinopathy) have been reported to be attributed essentially to the success of cuff reconstruction.

This study has some limitations; For instance, we did not subcategorize the patients based on types of rotator cuff pathologies. We also used subjective criteria, more than objective ones such as elbow strength in supination or flexion movements. The senior author performed all surgeries in both groups to minimize diversity of techniques between surgeons; however, this could be a limitation regarding the generalizability of the results. The study was performed in a single referral hospital; however, further work could be performed in multicenter hospitals with larger sample sizes to minimize bias. Our findings demonstrate similar effectiveness for both arthroscopic intraarticular and open subpectoral tenodesis. Therefore, it seems that there would be no differences in short- or mid-term results between these two techniques in treatment of rotator cuff tear associated with LHBT pathologies.
